# Increased thiamine transporter 1 RNA expression in the urinary sediment of type 1 diabetes patients with diabetic kidney disease

**DOI:** 10.1186/1758-5996-7-S1-A41

**Published:** 2015-11-11

**Authors:** Maria Beatriz Monteiro, Daniel Pereira Santos-Bezerra, Karina Thieme, Marcia Silva Queiroz, Marcia Nery, Maria Oliveira-Souza, Viktoria Woronik, Marisa Passarelli, Daniel Giannella-Neto, Ubiratan Fabres Machado, Maria Lúcia Corrêa-Giannella

**Affiliations:** 1 Faculdade de Medicina USP, São Paulo, Brazil

## Aims

Thiamine is reabsorbed by the transporters THTR1 and THTR2 (encoded by SLC19A2 and SLC19A3 genes, respectively) in kidney proximal tubule. Decreased plasmatic thiamine concentrations resulting from increased renal clearance was demonstrated in type 2 and type 1 diabetes (T1D) patients. Increased thiamine urinary excretion is an independent risk factor for renal function decline in T1D patients. To evaluate the participation of THTR1 and THTR2 in the pathogenesis of diabetic nephropathy (DN), we studied the expression of SLC19A2 and SLC19A3 in urinary sediment (US) and peripheral blood mononuclear cells (PBMC) in T1D. We also evaluated plasmatic thiamine concentrations to get insights about its status in our population.

## Materials and methods

Gene expression was evaluated by qPCR in 55 (US) and 161 (PBMC) participants sorted according to DN stage: absence of DN; incipient DN and overt DN, and according to estimated glomerular filtration rate (eGFR≥ or <60 mL/min/1.73m^2^); 26 healthy participants and 13 patients presenting focal and segmental glomerulosclerosis (FSGS) were included, respectively, as control group and as a group with non-diabetic nephropathy in the US experiments. Plasmatic thiamine concentrations were determined by HPLC.

## Results

In the US, SLC19A2 expression was higher in patients with (a) overt DN vs patients without DN (p=0.009) and to control participants (p=0.017) and (b) eGFR <60mL/min/1.73m2 vs eGFR ≥60mL/min/1.73m2 (p=0.01). ROC curve analyses demonstrated that SLC19A2 expression in the US was able to discriminate T1D patients according to DN status and to eGFR. Expression of SLC19A2 was negatively correlated with eGFR (p=0.01, r=-0.37) and positively correlated with plasma creatinine (p=0.002, r=0.43) in T1D patients. No association was found between SLC19A2 expression in US and the magnitude of eGFR decline after a median follow-up of 2 yrs. No difference was observed in PBMC SLC19A2 expression between T1D vs control participants. SLC19A3 mRNA was not detected in US and in PBMC of any participant. T1D and control participants showed thiamine plasmatic concentrations in the normal range, but lower concentrations were found in T1D vs control participants (p<0.0001).

## Conclusion

Normal thiamine plasmatic concentrations and absence of upregulation of SLC19A2 in PBMC suggest that upregulation of this gene in the US of patients with DN is not secondary to thiamine deficiency but to another underlying mechanism.

